# Azot expression in the *Drosophila* gut modulates organismal lifespan

**DOI:** 10.1080/19420889.2022.2156735

**Published:** 2022-12-28

**Authors:** Marisa M. Merino

**Affiliations:** Department of Biochemistry, Faculty of Sciences, University of Geneva, Geneva, Switzerland

**Keywords:** Cell competition, cell death, Azot, aging, gut, lifespan

## Abstract

Cell Competition emerged in *Drosophila* as an unexpected phenomenon, when confronted clones of fit *vs* unfit cells genetically induced. During the last decade, it has been shown that this mechanism is physiologically active in *Drosophila* and higher organisms. In *Drosophila*, Flower (Fwe) eliminates unfit cells during development, regeneration and disease states. Furthermore, studies suggest that Fwe signaling is required to eliminate accumulated unfit cells during adulthood extending *Drosophila* lifespan. Indeed, *ahuizotl* (*azot*) mutants accumulate unfit cells during adulthood and after physical insults in the brain and other epithelial tissues, showing a decrease in organismal lifespan. On the contrary, flies carrying three functional copies of the gene, unfit cell culling seems to be more efficient and show an increase in lifespan. During aging, Azot is required for the elimination of unfit cells, however, the specific organs modulating organismal lifespan by Azot remain unknown. Here we found a potential connection between gut-specific Azot expression and lifespan which may uncover a more widespread organ-specific mechanism modulating organismal survival.

## Main text

Cell Competition field emerged in *Drosophila* as an unexpected and intriguing phenomenon, when confronted clones of fit *vs* unfit cells genetically induced, in mosaic animals [1]. Surprisingly, unfit cells were eliminated from the tissue undergoing apoptosis, when they were present the fitter counterparts [1,2]. During the last decade it has been described that indeed, this mechanism is physiologically active in *Drosophila* and higher organisms [3–6]. Different studies show that organisms utilize Cell Competition in their entire lifetime by selecting the fittest cells, maintaining tissue and organ health [3–14]. Furthermore, we have also learnt about Cell Competition malfunctioning: tumoral cells hijack Cell Competition machineries to overcome host tissues [4,15–18].

Flower (Fwe) proteins have been described as regulators of competitive cell interactions in *Drosophila* and higher organisms [4–6,19–22]. The *fwe* gene in *Drosophila* encodes for three different Fwe isoforms; Fwe-Ubi, Fwe-LoseA and Fwe-LoseB [6,22,23]. Initially, Fwe role in Cell Competition was found by using competitive settings which were genetically induced in the developing *Drosophila* wing [21]. Further experiments showed then a primary physiological role for Flower in the developing retina, mediating the elimination of unwanted postmitotic neurons [6]. Fwe-dependent competition eliminates unfit cells by using tissue-specific fingerprints, in which different Fwe isoforms are required for unfit cell recognition and elimination [6,21].

Fwe proteins are multiple pass transmembrane proteins in which the C-terminus is extracellular [6,21,22]. Moreover, the Fwe proteins are also deployed in the tissue during wing development, coordinating growth and death phenomena [22,24]. The Decapentaplegic (Dpp) morphogen regulates organ growth in the *Drosophila* wing [25–31]. Dpp forms gradients in the target tissues and the ranges of these gradients keep proportional during organ growth [25,26] (*i.e*. they scale). In order to preserve this proportionality, there is a machinery which tunes the “size” of the Dpp gradient while the tissue grows keeping gradient and tissue proportional [25,26]. The scaling machinery consists of two proteins, Dally and Pentagone (Pent) and scaling homeostasis is ensured by molecular associations of these scaling factors with Fwe. In such a way that Dally/Pent scaling activity is tuned down by Fwe, while Fwe associated with the scaling partners inhibits its killing function [22,24,26,32,33]. Consistently, deleting Fwe extracellular sequences required for the association with the scaling partners is sufficient to trigger Fwe killing role [22,24].

**Figure 1.**
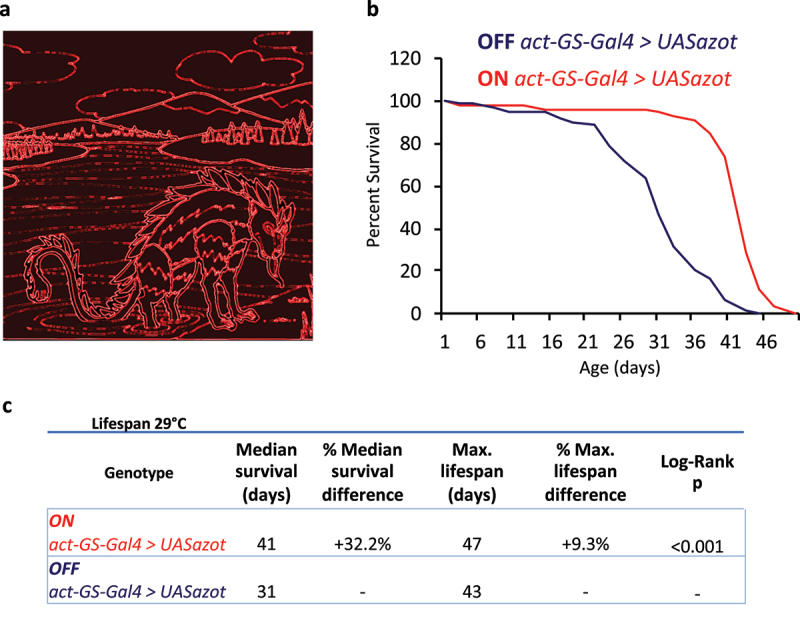
Ahuizotl creature and survival analysis after ubiquitous overexpression of Azot.

In *Drosophila*, Fwe eliminates unfit cells during development, regeneration and disease states during neurodegeneration [4–6,34,35]. Furthermore, studies suggest that Fwe signaling is required to eliminate unfit cells during adulthood extending *Drosophila* lifespan [5,34]. *Drosophila* mutants (*i.e. ahuizotl*) abrogating Fwe signal transduction pathway show developmental defects and compromised survival [5,34]. Ahuizotl, an EF-hand calcium binding protein has been characterized as a marker of unfit cells in Competition assays as well as in physiological scenarios [4,5,34]. Indeed, *ahuizotl* mutant flies accumulate unfit cells during adulthood, after physical insults and neurodegenerative setups in the brain and other epithelial tissues, showing a decrease in organismal lifespan [4,5,34]. On the contrary, flies carrying three functional copies of the gene, unfit cell culling seems to be more efficient and these flies show an increase in lifespan [5,34]. We named the gene *ahuizotl* after a mythological creature guardian of the lakes, said to protect the species from fishermen. *ahuizotl* (*azot*) name comes from the Aztec language Nahuatl and it means “spiny aquatic thing” [5,36,37] (Figure 1a).

**a.** Representation of Ahuizotl creature from Aztec mythology^36,37^ (modified from^58^). **b.** Survival plot, percent of survival vs time (days), comparing Azot overexpression (*UASazot*) at the organismal level (*act-GS-Gal4*) in RU-486-induced flies (ON) or RU-486-uninduced controls (OFF). **c.** Lifespan analysis at 29°C comparing control and Azot overexpressing flies at the organismal level. *act-GS-Gal4* driver was used for Azot overexpression at the organismal level. ON refers to RU-486-induced flies while OFF refers to RU-486-uninduced controls of the same genetic background. Genotypes, median survival (days), percentage of median survival difference, maximum lifespan (days), percentage of maximum lifespan difference and P values from log-rank test are shown for each genotype and condition.

During aging, Azot is required for the elimination of unfit cells, as previously described by using *azot* mutant flies [4,5]. However, specific organs modulating organismal lifespan by Azot remain unknown [4,5]. Is organ-specific expression of Azot sufficient to regulate organismal lifespan? To explore this question and eliminate genetic background effects, we used mifepristone (formerly known as RU-486) as inducing agent to activate *azot* transcription, taking advantage of the conditional RU486-dependent Gal4 system [38–41] (GeneSwitch (GS)). First, in order to overexpress Azot at the organismal level, we used *UASazot* driven by the ubiquitous inducible driver *act-GS-Gal4*. Under these conditions, RU486-induced flies ubiquitously overexpressing Azot (ON *act-GS-Gal4 > UASazot*) show an increase in survival compared to control RU486-uninduced siblings (OFF *act-GS-Gal4 > UASazot*) (Figure 1b,c and Table 1,2). These data are consistent with previous studies at the organismal level, where by increasing the number of functional copies of *azot*, flies eliminate more efficiently unfit cells and show an increase in lifespan compared to control ones [4,5].

**Table 1.** Alleles.

Alleles	Details
*5966-GS*-*Gal4*	[38–40]
*act-GS-Gal4*	[38–40]
*UASazot*	[5]
*azotRNAi*	VDRC #18166

**Table 2.** Detailed genotypes.

Figure	Genotype
1b	*act-GS-Gal4 > UASazot* (ON) *act-GS-Gal4 > UASazot* (OFF)
1c	*act-GS-Gal4 > UASazot* (ON) *act-GS-Gal4 > UASazot* (OFF)
2a	*5966-GS-Gal4 > UASazot* (ON) *5966-GS-Gal4 > UASazot* (OFF)
2b	*5966-GS-Gal4 > azotRNAi* (ON) *5966-GS-Gal4 > azotRNAi* (OFF)
2c	*5966-GS-Gal4 > UASazot* (ON) *5966-GS-Gal4 > UASazot* (OFF) *5966-GS-Gal4 > azotRNAi* (ON) *5966-GS-Gal4 > azotRNAi* (OFF)

**Figure 2.**
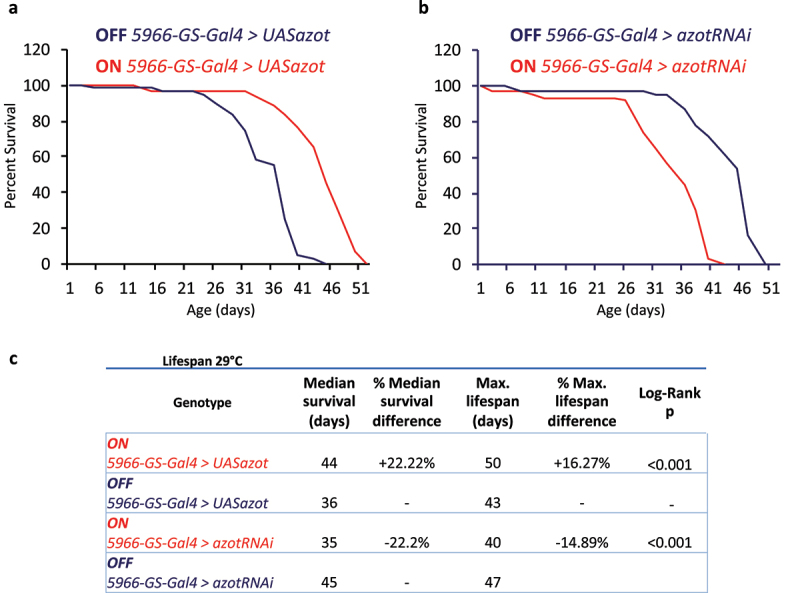
Survival analysis after organ-specific overexpression/downregulation of Azot.

To test possible lifespan phenotypes overexpressing Azot in organ-specific manner, we focus on the *Drosophila* gut. In recent years, the link between intestinal homeostasis and organismal lifespan has been described by a great number of studies in the field, supporting that the intestine is a key organ regulating organismal lifespan [42–47]. We therefore targeted the gut enteroblast/enterocyte (EB/EC) cells by using the RU486-inducible *5966-GS-Gal4* driver [44,48]. Interestingly, RU486-induced flies targeting Azot overexpression into the EB/EC cells (ON *5966-GS-Gal4 > UASazot*) show increased survival compared to the RU486-uninduced control siblings (OFF *5966-GS-Gal4 > UASazot*) (Figure 2a,c and Table 1,2). On the contrary, RU486-induced flies downregulating Azot in the EB/EC cells (ON *5966-GS-Gal4 > azotRNAi*) show a decrease in survival compared to the RU486-uninduced control flies (OFF *5966-GS-Gal4 > azotRNAi*) (Figure 2b,c and Tables 1, 2).

**a.** Survival plot, percent of survival vs time (days), comparing Azot overexpression (*UASazot*) upon EB/EC-specific targeting (*5966-GS-Gal4*) in RU-486-induced flies (ON) or RU-486-uninduced controls (OFF). **b.** Survival plot, percent of survival vs time (days), comparing Azot downregulation (*azotRNAi*) upon EB/EC-specific targeting (*5966-GS-Gal4*) in RU-486-induced flies (ON) or RU-486-uninduced controls (OFF). **c.** Lifespan analysis at 29°C comparing Azot overexpression (*UASazot*) and Azot downregulation (*azotRNAi*) in the gut-specific EB/EC cells. *5966-GS-Gal4* driver was used to specifically target EB/EC cells. ON refers to RU-486-induced flies while OFF refers to RU-486-uninduced controls of the same genetic background. Genotypes, median survival (days), percentage of median survival difference, maximum lifespan (days), percentage of maximum lifespan difference and P values from log-rank test are shown for each genotype and condition.

**Figure 3.**
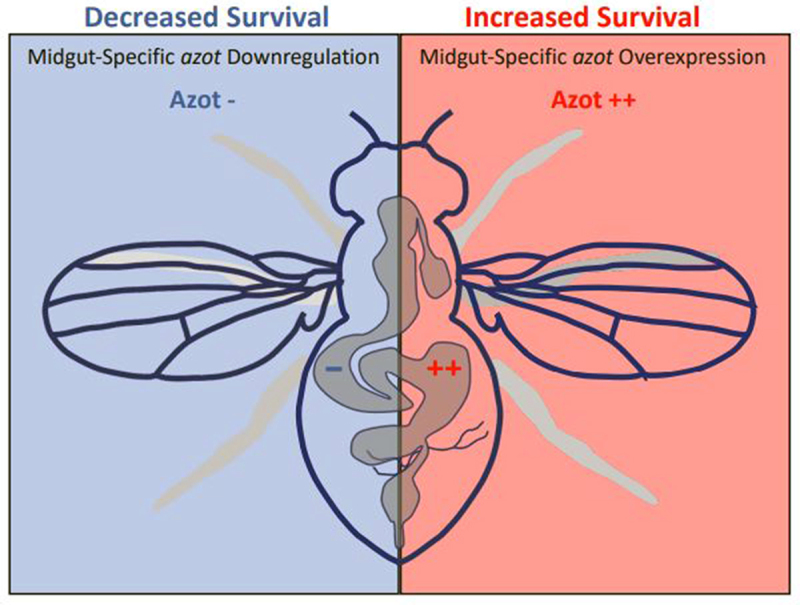
Schematic model comparing survival when modulating Azot expression in organ-specific manner in the EB/EC cells.

Thus, these data suggest that Azot expression in organ-specific manner (*i.e*. in the gut) is sufficient to modulate *Drosophila* lifespan (Figure 3 and Tables 1,2). In the case of the *Drosophila* gut, we might speculate that these data could underlie organ-specific effects maintaining functionality longer by enhancing the digestive/barrier function [40,42–44,49–52], probably improving tissue health by boosting the elimination of unfit cells [4,5]. Alternatively and/or synergistically, these data might reflect the central role of the *Drosophila* gut regulating systemic homeostasis and organ-to-organ communication [40,42–44,46,47,49–51,53–55]. Here we found a potential connection between gut-specific Azot expression and lifespan which may uncover a more widespread organ-specific mechanism modulating organismal survival.

Azot specific overexpression (RU-induced; *5966-GS-Gal4 >UASazot*) in the EB/EC cells increases organismal survival (Azot ++, red rectangle). Conversely, when downregulating Azot expression (RU-induced; *5966-GS-Gal4 >azotRNAi*) in the midgut EB/EC cells, flies show a decreased survival (Azot -, blue rectangle).

## Material and methods

### Lifespan analyses

*Drosophila* lines and crosses were kept on standard cornmeal fly food vials. Cohorts of 100 females flies (1–3 days old) per genotype were collected and kept at 29°C under light-dark cycle. Deaths were scored at regular intervals (three times per week) and surviving flies were transferred to new vials. GS system was used to minimize background effects when comparing different *Drosophila* genotypes [38–40]. For *UAS* induction (RU486-induced), the stock solution of RU486 (Mifepristone, Sigma, prepared in 80% ethanol) was diluted in Mili-Q water to a final concentration of 100 μM. Then, 300 μL of the diluted solution was added to the surface of the fly food and allowed to dry at room temperature for 48 h. Similarly control flies (RU486-uninduced) received vehicle control [34,56].

### Statistical analyses

To carry out statistical analyses and quantify lifespan values we used the application OASIS 2. The log-rank test was used to analyze differences between survival curves and determine P values [57].
